# Supplementary material for: Nonlinear relationship between platelet count and 30-day in-hospital mortality in intensive care unit stroke patients: a multicenter retrospective cohort study
Source: Front Neurol. 2024 Apr 24;15:1374159. doi: 10.3389/fneur.2024.1374159 (PMC11076867; doi:10.3389/fneur.2024.1374159)
Supplement: Supplementary file 1 [file Data_Sheet_1.pdf]

Nonlinear relationship between platelet count and 30-day in-hospital mortality in ICU stroke patients: a multicenter retrospective cohort study

Table S1. Collinearity diagnostics steps

Variable	VIF Step 1
Sex	1.1
Ethnicity	1
Age(years)	1.3
BMI(kg/m^2)	1
Hb(g/dl)	1.2
Scr(mg/dl)	1.2
APACHE-IV score	1.7
ARF	1.5
AF	1.1
ACS	1.1
CHF	1.1
CKD	1
COPD	1.1
Diabetes mellitus	1.2
GB	1
Hypertension	1.2
Sepsis	1.2
Anti-platelet	1.1
Anticoagulant	1.1
Glucocorticoid	1.1
Carbapenems	1.1
Cephalosporins	1.2
Vancomycin	1.3
Mechanical ventilation	1.6

Abbreviation: VIF: variance inflation factor; VIF = 1/ (1-R^2).

Note: The variables with VIF>5 will be regarded as collinear variables and cannot be included in the multiple regression model

BMI, body mass index; Hb, hemoglobin; Scr, creatinine; APACHE-IV score, Acute Physiology and Chronic Health Evaluation–IV score; ARF, acute respiratory failure; AF, atrial fibrillation; ACS, acute coronary syndrome; CHF, congestive heart failure; CKD, chronic kidney disease; COPD, chronic obstructive pulmonary disease; GB, gastrointestinal bleeding; Mortality, 30-day in-hospital mortality

Table S2 Influencing factors of 30-day in-hospital mortality using univariate regression analysis

Variable	Statistic	OR95%CI	*P*
Sex			
male	4268 (51.99%)	1.0	
Female	3941 (48.01%)	1.01 (0.89, 1.14)	0.8774
Ethnicity			
Caucasian	6094 (74.24%)	1.0	
African American	1056 (12.86%)	0.89 (0.73, 1.09)	0.2612
Hispanic	400 (4.87%)	1.26 (0.96, 1.65)	0.0980
Asian	172 (2.10%)	1.38 (0.93, 2.04)	0.1145
Other/unknown	487 (5.93%)	1.25 (0.97, 1.60)	0.0818
Age(years)	66.95 ± 14.91	1.01 (1.01, 1.02)	<0.0001
BMI(kg/m^2)	27.95 ± 8.50	0.99 (0.98, 1.00)	0.0366
Hb(g/dl)	12.75 ± 2.32	0.94 (0.91, 0.96)	<0.0001
Scr(mg/dl)	1.27 ± 1.29	1.12 (1.07, 1.16)	<0.0001
APACHE-IV score	55.67 ± 24.92	1.04 (1.04, 1.04)	<0.0001
Platelet count	222.57 ± 75.49	0.99 (0.99, 0.99)	<0.0001
**Comorbid conditions**			
ARF			
No	6682 (81.40%)	1.0	
Yes	1527 (18.60%)	4.51 (3.94, 5.16)	<0.0001
AF			
No	7156 (87.17%)	1.0	
Yes	1053 (12.83%)	1.37 (1.15, 1.62)	0.0004
ACS			
No	7835 (95.44%)	1.0	
Yes	374 (4.56%)	1.87 (1.45, 2.40)	<0.0001
CHF			
No	7778 (94.75%)	1.0	
Yes	431 (5.25%)	1.83 (1.44, 2.31)	<0.0001
CKD			
No	8080 (98.43%)	1.0	
Yes	129 (1.57%)	1.27 (0.80, 2.01)	0.3184
COPD			
No	7841 (95.52%)	1.0	
Yes	368 (4.48%)	1.52 (1.17, 1.99)	0.0019
Diabetes mellitus			
No	7108 (86.59%)	1.0	
Yes	1101 (13.41%)	1.41 (1.19, 1.66)	<0.0001
GB			
No	7980 (97.21%)	1.0	
Yes	229 (2.79%)	1.31 (0.92, 1.85)	0.1288
Hypertension			

No	5569 (67.84%)	1.0	
Yes	2640 (32.16%)	0.98 (0.85, 1.12)	0.7255
Sepsis			
No	7693 (93.71%)	1.0	
Yes	516 (6.29%)	2.63 (2.14, 3.22)	<0.0001
**Treatment**			
Anti-platelet			
No	7711 (93.93%)	1.0	
Yes	498 (6.07%)	0.99 (0.76, 1.28)	0.9125
Anticoagulant			
No	8044 (97.99%)	1.0	
Yes	165 (2.01%)	0.99 (0.64, 1.55)	0.9756
Glucocorticoid			
No	7873 (95.91%)	1.0	
Yes	336 (4.09%)	1.82 (1.39, 2.37)	<0.0001
Carbapenems			
No	8158 (99.38%)	1.0	
Yes	51 (0.62%)	2.11 (1.12, 3.97)	0.0207
Cephalosporins			
No	7868 (95.85%)	1.0	
Yes	341 (4.15%)	1.38 (1.04, 1.83)	0.0244
Vancomycin			
No	7882 (96.02%)	1.0	
Yes	327 (3.98%)	2.56 (2.00, 3.29)	<0.0001
Mechanical ventilation			
No	6041 (73.59%)	1.0	
Yes	2168 (26.41%)	7.27 (6.35, 8.31)	<0.0001

BMI, body mass index; Hb, hemoglobin; Scr, creatinine; APACHE-IV score, Acute Physiology and Chronic Health Evaluation–IV score; ARF, acute respiratory failure; AF, atrial fibrillation; ACS, acute coronary syndrome; CHF, congestive heart failure; CKD, chronic kidney disease; COPD, chronic obstructive pulmonary disease; GB, gastrointestinal bleeding.

Table S3 Relationship between platelet and 30-day in-hospital mortality in different models

Exposure	Crude model (OR, 95%CI, P)	Model I (OR, 95%CI, P)	Model II (OR, 95%CI, P)
Platelet count	0.99 (0.99, 0.99) <0.0001	0.99 (0.99, 0.99) <0.0001	0.99 (0.99, 0.99) <0.0001
Platelet count (quartile)			
Q1	Ref.	Ref.	Ref.
Q2	0.61 (0.51, 0.72) <0.0001	0.61(0.51, 0.73) <0.0001	0.70 (0.57, 0.85) 0.0004
Q3	0.68 (0.57, 0.81) <0.0001	0.69 (0.58, 0.82) <0.0001	0.77 (0.64, 0.94) 0.0102
Q4	0.64 (0.53, 0.75) <0.0001	0.67 (0.56, 0.80) <0.0001	0.65 (0.53, 0.79) 0.0002
P for trend	<0.0001	<0.0001	0.0001

Abbreviations: CI, confidence interval.

Model I adjusted for age, sex, and ethnicity.

Model II adjusted for adjusted age, sex, and ethnicity, BMI, Hb, Scr, APACHE-IV score, ARF, AF, ACS, CHF, CKD, COPD, Diabetes mellitus, GB, hypertension, sepsis, anti-platelet, anticoagulant, glucocorticoid, carbapenems, cephalosporins, vancomycin, and mechanical ventilation
